# Quadratus lumborum block for postoperative pain management in patients undergoing total hip arthroplasty: a systematic review and meta-analysis

**DOI:** 10.1177/11207000221111309

**Published:** 2022-07-17

**Authors:** Aaron M Gazendam, Meng Zhu, Luc Rubinger, Yaping Chang, Steve Phillips, Mohit Bhandari

**Affiliations:** 1 OrthoEvidence, Burlington, ON, Canada; 2 Division of Orthopaedic Surgery, Department of Surgery, McMaster University, Hamilton, ON, Canada

**Keywords:** Perioperative, quadratus lumborum block, total hip arthroplasty

## Abstract

**Background:**

The use of quadratus lumborum nerve blocks (QLB) for pain control following elective total hip arthroplasty (THA) has increased substantially in recent years. The objective of this systematic review and meta-analysis was to compare outcomes from randomised controlled trials (RCTs) utilising QLBs following elective THA.

**Methods:**

MEDLINE, EMBASE, and Cochrane databases were searched for RCTs perioperative QLBs for THA. Quantitative synthesis was conducted for pain scores, opioid consumption and adverse events.

**Results:**

A total of 7 RCTs with 429 patients undergoing THA were included. No differences in pain scores were demonstrated between QLBs and control interventions. Subgroup analysis demonstrated no differences between QLBs and sham procedures or active comparators. No differences in postoperative opioid consumption between QLB and control interventions was found. In trials reporting adverse events, they were rare and similar between groups. Overall, the certainty of the evidence was graded as low or very low

**Conclusions:**

The current literature suggests that a QLB for THA does not reduce pain or opioid consumption compared to sham or active comparators.

## Introduction

Total hip arthroplasty (THA) is the gold standard for management of end-stage osteoarthritis and offers tremendous improvements in function and quality of life. However, THA is a major surgical intervention and is associated with significant pain in the immediate postoperative setting. Given the ongoing opioid epidemic and negative outcomes associated with prolonged opioid use following THA, nonopioid adjuncts are an active area of research.^1–3^

Multimodal analgesia protocols have been introduced in recent years in attempts to improve pain control and reduce opioid consumption. These protocols aim to provide relief through targeting different pain pathways and comprise of non-opioid oral analgesics, cryotherapy, periarticular injections and regional nerve blocks.^4^ An ideal regional nerve block provides pain relief to the surgical area while preserving motor function to allow for early postoperative mobilisation, especially important with the increased utilisation of same-day discharge THA care pathways.^5^ Early studies examined the use of femoral nerve or lumbar plexus blocks following THA but these techniques have demonstrated high rates of motor weakness postoperatively.^6,7^

The use of quadratus lumborum nerve blocks (QLB) have increased dramatically in hip and pelvis surgery in recent years. First described in 2007 by Blanco^8^, the QLB is a fascial plane block that aims to anaesthetise the thoracolumbar nerves.^8,9^ There are 4 contemporary techniques that are described, each named according to the needle tip position relative to the quadratus lumborum muscle: the lateral, posterior, anterior and intramuscular approaches.^10,11^ A recent meta-analysis of 42 randomised controlled trials (RCTs) by Uppal et al.^12^ demonstrated that QLBs provided pain relief compared to placebo in patients undergoing abdominal wall and hip surgery. The review was limited by the heterogeneity of patients and procedures included. Another review by Jin et al.^13^ did try and address this heterogeneity by performing a subgroup analysis on only 2 included studies in the orthopaedic hip surgery population. Likewise, the review by Korgvee et al.^14^ performed a subgroup analysis on those undergoing hip surgery but failed to analyse the arthroplasty population in isolation.

With at least 7 RCTs being published since 2020, QLB for postoperative pain management in patients undergoing THA is an area of interest. To our knowledge, there are no reviews synthesising the data to examine the impact of QLBs in the specific patient population of those undergoing THA. Thus, a systematic review and meta-analysis of RCTs to determine the efficacy of QLB in providing postoperative analgesia for patients undergoing THA compared to placebo or other analgesic techniques was carried out.

## Methods

This systematic review was conducted in accordance with the PRISMA (Preferred Reporting Items for Systematic Reviews and Meta-Analyses) guidelines for conducting and reporting systematic reviews.^15^ The study protocol was registered prospectively on The International Prospective Register of Systematic Reviews (PROSPERO #CRD42021275225).

### Literature search

The online databases MEDLINE, Embase, Web of Science, and Cochrane Controlled Register of Trials (CENTRAL) were searched from database inception to 24 August 2020, for literature pertaining to the use of QLB and THA. Related systematic reviews and reference lists were also searched for additional eligible studies. A full search strategy is available in Supplemental Material Table 1.

### Study screening

Studies identified during the search were screened at the title and abstract, as well as full text stage by 2 blinded reviewers (MZ and YC), in duplicate, using the online software Rayyan QCRI (2010, Qatar Computing Research Institute, Doha, Qatar).^16^ Any discrepancies at the title and abstract stage were resolved through consensus. At the full text stage of screening any discrepancies were discussed and resolved by consensus between the reviewers.

### Assessment of study eligibility

The inclusion and exclusion criteria were defined *a priori*. Inclusion criteria were: (1) randomised controlled trials; (2) patients undergoing THA regardless of surgical approach used; (3) utilised a perioperative QLB compared to sham or active comparator; and (4) studies were published in the English language. Studies were excluded if: (1) non-randomised; (2) THA for fracture; (3) involved surgical interventions other than THA; (4) papers without extractable primary data.

### Data extraction

2 reviewers (MZ & YC) extracted data from included studies into a collaborative spreadsheet (Google, California, USA) designed *a priori*. Extracted data included patient and study characteristics, QLB technique, comparator techniques, pain outcomes, opioid consumption, adverse events or complications.

### Study appraisal

The risk of bias in the included randomised controlled trials was assessed using the Revised Cochrane Risk of Bias Tool (Cochrane Risk of Bias 2.0).^17^ The overall quality of the evidence was evaluated using the GRADE approach (Grading of Recommendations, Assessment, Development and Evaluations).^18^

### Statistical analysis

Quantitative synthesis was conducted using Review Manager 5 (RevMan) for pain outcomes measured by the visual analogue scale (VAS) and functional outcomes. As per the guidelines set out by the GRADE all scores for each measured outcome were converted to a common scale.^18^ Pain scales included the VAS and the Numeric Rating Scale (NRS).^19,20^ All pain scores were converted to a normalised scale with a range between 0–10. After data conversion, a mean difference (MD) with 95% confidence intervals (CI) were calculated and reported accordingly. Subgroup analyses were performed based on the type of QLB block (posterior or transmuscular) and based on the type of comparator (sham vs. active). Narrative summary of evidence was also collected. A *p*-value of <0.05 was considered statistically significant for all outcomes.

## Results

### Literature search

**Figure 1.**
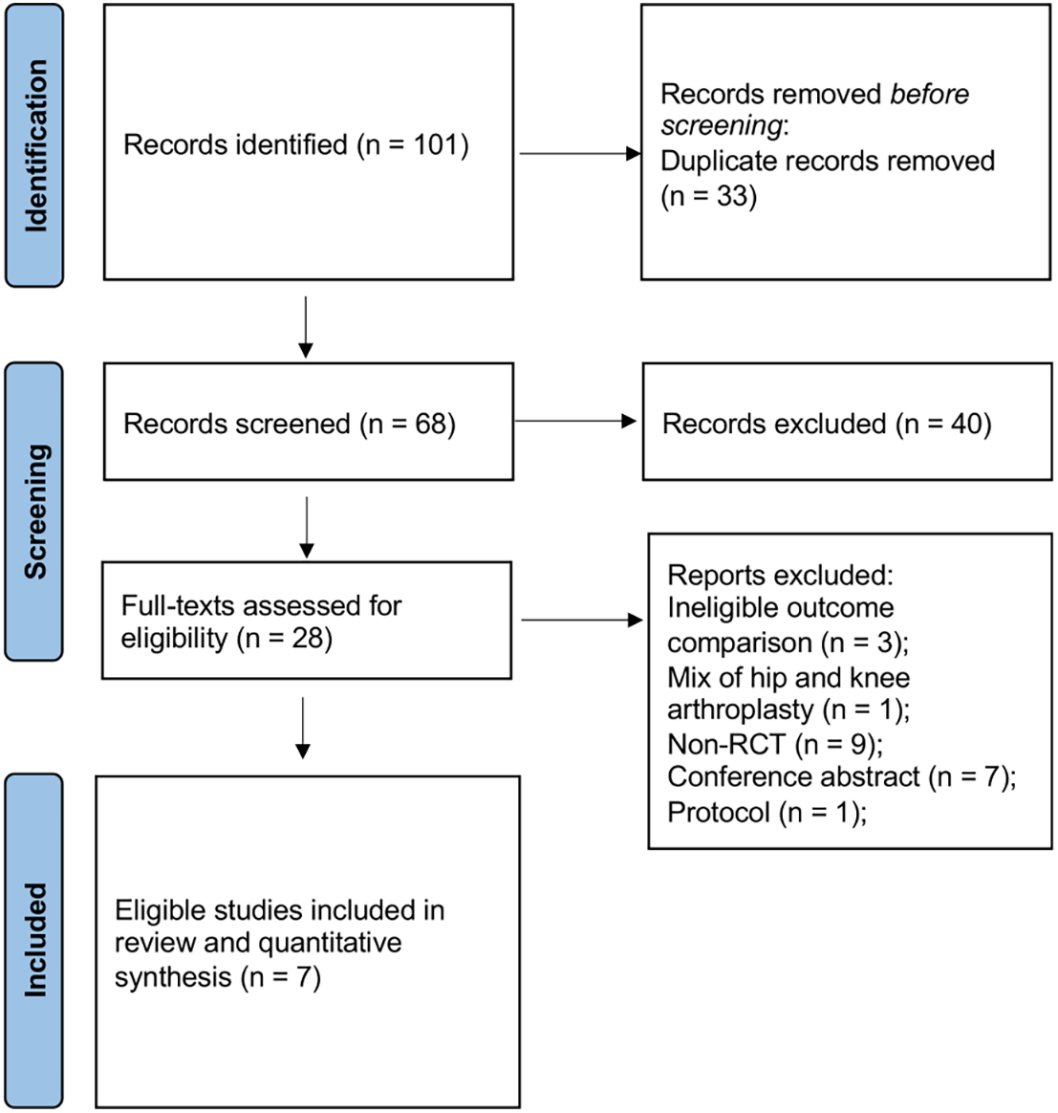
PRISMA flow diagram.

The initial search yielded 101 unique studies. Systematic screening and assessment of eligibility resulted in 7 full text studies that met the inclusion criteria.^10,21–26^ A PRISMA flow diagram detailing the search and screening process is displayed in Figure 1.

### Study quality

All 7 of the included studies were published from 2020 to 2021 (Table 1). In terms of risk of bias assessment (Appendix), all included studies showed a low risk of bias in random sequence generation, allocation concealment, blinding of outcome assessment, and selective reporting. 3 studies did not mask participants.^22,24,26^ 3 RCTs did not provide detailed information on criteria for anaesthesiologist’s participation or expertise.^10,21,26^ Overall, the certainty of the evidence was graded as low or very low (Appendix).

**Table 1.** Characteristics of included studies.

Study	Country	No. of patients at randomisation	Age [years, mean (SD)]	Intervention	Control
Abduallah et al.^21^	Egypt	60	Intervention: 67.90 (4.8); Control: 66.43 (3.89)	Ultrasound-guided transmuscular QLB at the end of THA	Sham procedure
Aoyama et al.^22^	Japan	30	Intervention: 70 (9); Control: 67 (11)	Ultrasound-guided anterior QLB before general anaesthesia and immediately after THA	Femoral nerve block
Brixel et al.^10^	France	100	Intervention: 68 (range: 59–72); Control: 65 (59–72)	Ultrasound-guided posterior QLB before general anaesthesia	Sham procedure
He et al.^23^	China	88	Intervention: 66 (7); Control: 67 (8)	Ultrasound-guided transmuscular QLB before THA	Sham procedure
Hu et al.^24^	China	80	Intervention: 58.78 (12.16); Control: 55.73 (13.45)	Ultrasound-guided transmuscular QLB before general anaesthesia + local analgesia	Local analgesia only
Nassar et al.^26^	Egypt	38	Intervention: 54 (16); Control: 47 (17.6)	Ultrasound-guided transmuscular QLB before anaesthesia	Fascia iliaca block
Gutierrez et al.^25^	USA	50	Intervention: 68.6 (11.8); Control: 65.7 (9.8)	Ultrasound-guided transmuscular QLB before anaesthesia	Lumbar plexus block

SD, standard deviation; QLB, quadratus lumborum block; THA, total hip arthroplasty.

### Patient characteristics

There was a total of 429 patients analysed in the 7 included studies.^10,21–26^ All patients underwent primary, unilateral THA. Of these, 214 received a QLB and 215 received a control intervention. With respect to the type of QLB performed, 6 RCTs evaluated the anterior or transmuscular approach (*n* *=* 164) and 1 study evaluated the posterior QLB (*n* *=* 50). 3 studies utilised a sham injection as a comparator (*n* *=* 122), 1 study utilised local anaesthetic infiltrate (*n* *=* 40), 1 utilised a femoral nerve block (*n* *=* 11), 1 a fascial iliac block (*n* *=* 19) and 1 utilised a lumbar plexus block (*n* *=* 23).

### Pain outcomes

**Figure 2.**
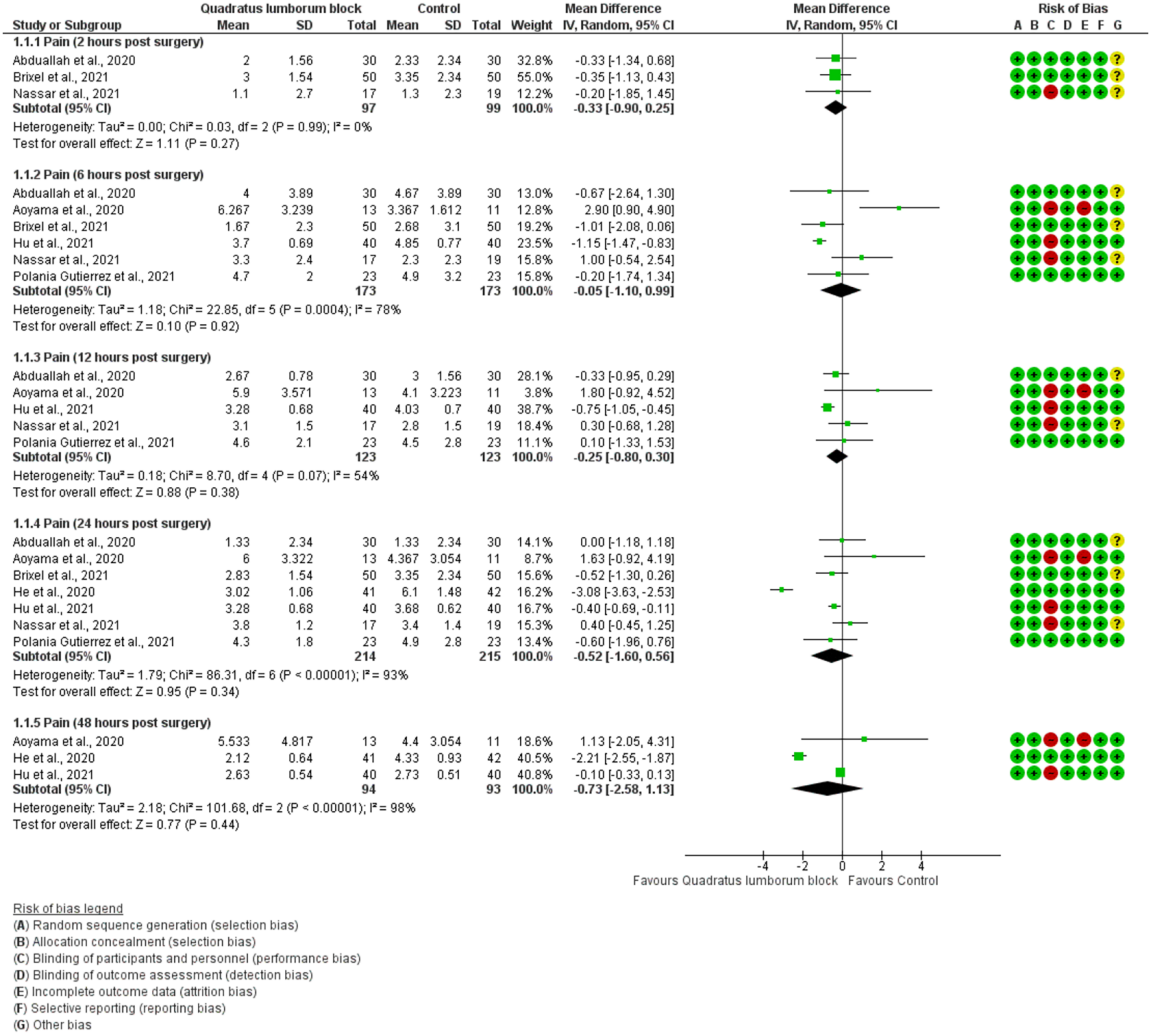
Forest plots of pain on a normalised scale (0–10).

All 7 included studies reported postoperative pain and utilised a VAS or NRS pain score. Pain was evaluated at 2,6, 12, 24 and 48 hours postoperatively. As shown in Figure 2, there was no significant differences in pain scores found between QLB and control (including sham and active comparators) at any timepoint postoperatively. When evaluating the anterior/transmuscular QLB versus control, no significant differences in pain scores were found at 2 hours (mean difference [MD] −0.29; 95% confidence interval [CI]: −1.15 to 0.56], 6 hours [MD 0.24; 95% CI, −1.16 to 1.65), and 24 hours (MD −0.50; 95% CI, −1.80 to 0.80) post THA. In the 1 study examining the posterior QLB, no differences in pain scores were found at 2, 6 and 24 hours postoperatively.

A subgroup analysis was also performed based on the type of the control intervention (nerve block or sham). Effect estimates showed no statistical significance for either control groups (vs. sham: MD −1.24; 95% CI, −3.29 to 0.81; vs. nerve block: MD 0.22; 95% CI, −0.69 to 1.13) (forest plot not shown). Hu et al.^24^ was the only RCT comparing QLB versus local infiltration analgesia and showed that QLB significantly relieved pain at 6 hours (MD −1.15; 95% CI, −1.47 to −0.83), 12 hours (MD −0.75; 95% CI, −1.05 to −0.45), and 24 hours post-surgery (MD −0.40; 95% CI, −0.69 to −0.11) (Figure 2).

### Morphine consumption

**Figure 3.**
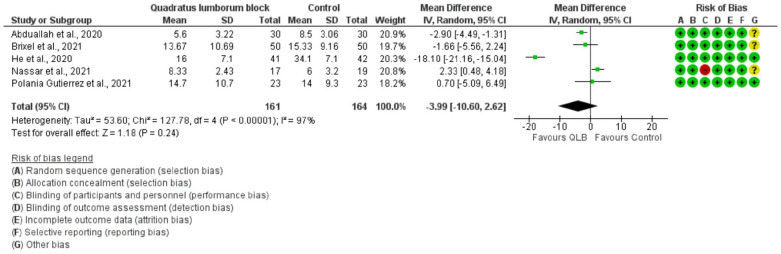
Forest plot demonstrating opioid consumption (mg, 24 hours after surgery).

5 of the included studies reported postoperative opioid consumption displayed as intravenous morphine consumption 24 hours postoperatively. There was no significant difference in morphine consumption at 24 hours postoperatively between QLB and control groups (Figure 3).

Subgroup analysis of the type of control group (sham or nerve block) and opioid consumption was performed. No significant difference in opioid consumption between QLB and sham procedures was found (MD −7.56 mg; 95% CI, −17.56 to 2.45). When comparing QLB to other nerve blocks (FIB and LPB), the nerve blocks in the control group significantly reduced opioid consumption at 24 hours post THA (MD 2.18 mg; 95% CI, 0.42–3.94), compared to QLB.

### Adverse events

**Figure 4.**
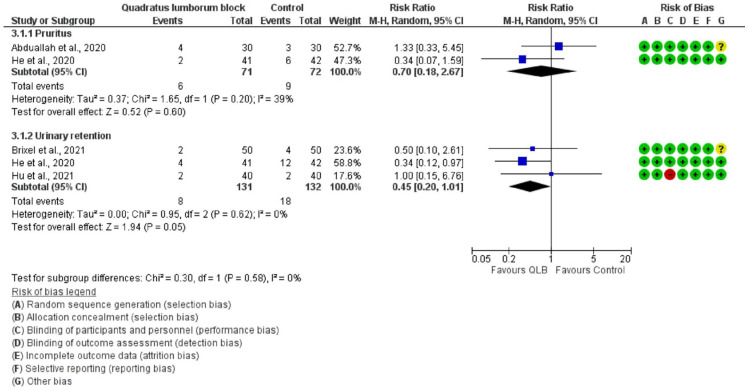
Incidence of pruritus and urinary retention.

No significant differences were found between QLB versus control in the incidence of complications namely pruritus or urinary retention (Figure 4). Aoyama et al.^22^ and Polania Gutierrez et al.^25^ reported no severe complications for both QLB and control groups.

## Discussion

To our knowledge, this is the first systematic review and meta-analysis specifically evaluating QLB in the THA patient population. The key finding of this review is that in the perioperative management of patients undergoing primary elective THA, regional anaesthesia in the form of QLB does not provide superior pain relief or reduce morphine consumption compared to placebo, sham or other nerve blocks. Only 1 study showed positive QLB results; when compared to local infiltration analgesia.^24^ With that said, this review showed that QLBs do not cause significantly more complications and adverse effects relative to the comparable treatments.

Recently, 4 systematic reviews and meta-analyses investigating QLBs have been published.^12–14,27^ According to these reviews, utilisation of QLB compared to sham or placebo, reduces postoperative opioid consumption after abdominal, pelvic and hip surgery. In the review performed by Jin et al.,^13^ subgroup analyses were carried out according to the type of surgery. They only isolated 2 studies concentrating on hip surgery (not specific to elective arthroplasty), with “both reporting significant opioid sparing effect, however, with considerable heterogeneity”.^13^ The recent meta-analysis by Uppal et al.^12^ also demonstrated that QLBs provided pain relief compared to placebo in patients undergoing abdominal wall and hip surgery, with limitations due to the heterogeneity of patients and procedures included. In the review of Kim et al.,^27^ lower pain scores were reported in QLB groups but no subgroup analyses were performed to stratify based on surgical procedure. Conversely, the review by Korgvee et al.^14^ categorised QLB based on laterality compared to the operative side (or bilateral) and stratified their analysis, similarly to this review, based on the approach used for the QLB and comparator treatment utilised (i.e. no block/placebo, other peripheral block and central block studies). Their results also analysed pain outcomes based on the type of surgery performed, with inclusion of 4 ‘hip surgery’ studies showing efficacy of the QLB in reducing postoperative narcotic use. While Korgvee et al.^14^ stated that the QLB had a favourable side effect profile, that was also supported by the lower risk of postoperative nausea and vomiting (PONV) in QLB groups, their primary results, as with the other reviews, are in discordance with ours.

The feverishness with which the medical community is investigating the QLB is evident with 7 RCTs investigating QBL the hip arthroplasty population have been published in the past 2 years. Furthermore, the aforementioned recently published meta-analyses that have all concluded the relative efficacy of these QLB procedures in reducing postoperative pain and opioid burden in those undergoing pelvic, abdominal and hip surgeries. However, the discordance of their results with the findings of this review points to the importance of analysing a homogeneous surgical patient population when performing potentially practice-changing meta-analytic reviews. This review herein provides a meta-analysis of the effect of QLB on those undergoing THA, a singular surgical procedure that was only performed in an elective population. This homogeneity of the population analyses provides a solid foundation on which to base the clinical decision that QLB are not currently indicated for the primary aim of decreasing pain or opioid burden in this patient population. This review is further strengthened by its rigorous methodology, adherence to the PRISMA guidelines and the inclusion of only level 1 evidence.

This study has several limitations. Firstly, it is limited by the quality of the current available literature. The included studies were relatively small, single-centre studies that may be underpowered to demonstrate differences between groups in the primary and secondary outcomes. Secondly, there was heterogeneity within the control groups used to compare QLB against, as both active and sham comparators were included. However, subgroup analysis demonstrated similar results. Further to this point on heterogeneity, the surgical patient populations were heterogeneous in the sense that all surgical approaches for THA were included. Given that different dermatomes and myotomes are incised with the various surgical approaches, the nervous block provided by the QLB may favour 1 specific approach. As well, none of the included studies evaluated the impact of the QLB on postoperative mobility as an outcome. The meta-analysis performed by Korgvee et al.^14^ was based on 7 studies (only 1 study was orthopaedic in nature) and found that pain scores reductions during movement with QLB resembled the results at rest. With the continuing adoption of same-calendar-day total joint replacement, the motor blockade of a chosen anaesthetic treatment and its impact on pain during mobility, can have significant implications for the patient in terms of need for admission, and costing of the total care provided.

Another limitation is that data on anaesthesia related complications such as PONV were not widely collected in the included studies and thus could not be synthesised. PONV are 2 very well described side effects of opioids.^28^ The frequency and intensity of PONV are lowered in proportion to the reduction in intra- and postoperative opioid consumption. Our review was unable to document changes in intraoperative opioid consumption, antiemetic utilisation or PONV, which are all appropriate considerations in selecting a nervous system block perioperatively.

## Conclusion

In patients undergoing primary elective THA, perioperative QLB does not provide superior pain relief or reduce opioid consumption compared to sham nerve blocks or active comparators. These results are discordant with other recent meta-analyses that concluded that QLBs were significantly effective in the hip surgery population. Further studies are needed to discern if there are specific THA patients who may benefit from a certain type of QLB perioperatively, such as those when a certain surgical approach is used, a mobility preserving nervous block is required, a certain QLB approach is utilised, or otherwise. With the continued increase in study in the field of QLB, a unifying nomenclature and method of administration should be adopted so that like procedures can be truly compared.

## Supplemental Material

sj-pdf-1-hpi-10.1177_11207000221111309 – Supplemental material for Quadratus lumborum block for postoperative pain management in patients undergoing total hip arthroplasty: a systematic review and meta-analysis Click here for additional data file. Supplemental material, sj-pdf-1-hpi-10.1177_11207000221111309 for Quadratus lumborum block for postoperative pain management in patients undergoing total hip arthroplasty: a systematic review and meta-analysis by Aaron Gazendam, Meng Zhu, Luc Rubinger, Yaping Chang, Steve Phillips and Mohit Bhandari in HIP International

sj-tiff-2-hpi-10.1177_11207000221111309 – Supplemental material for Quadratus lumborum block for postoperative pain management in patients undergoing total hip arthroplasty: a systematic review and meta-analysis Click here for additional data file. Supplemental material, sj-tiff-2-hpi-10.1177_11207000221111309 for Quadratus lumborum block for postoperative pain management in patients undergoing total hip arthroplasty: a systematic review and meta-analysis by Aaron Gazendam, Meng Zhu, Luc Rubinger, Yaping Chang, Steve Phillips and Mohit Bhandari in HIP International
