# Supplementary material for: Quadratus lumborum block for postoperative pain management in patients undergoing total hip arthroplasty: a systematic review and meta-analysis
Source: Hip Int. 2022 Jul 17;33(5):850–7. doi: 10.1177/11207000221111309 (PMC10486167; doi:10.1177/11207000221111309)
Supplement: sj-pdf-1-hpi-10.1177_11207000221111309 – Supplemental material for Quadratus lumborum block for postoperative pain management in patients undergoing total hip arthroplasty: a systematic review and meta-analysis [file sj-pdf-1-hpi-10.1177_11207000221111309.pdf]

Search Strategy

Ovid MEDLINE

1. Arthroplasty, Replacement, Hip/
2. (total hip arthroplasty or total hip replacement or THA or THR).mp.
3. quadratus lumborum block.mp.
4. 1 or 2
5. 3 and 4

Ovid EMBASE

1. total hip replacement/
2. (total hip arthroplasty or total hip replacement or THA or THR).mp.
3. quadratus lumborum block.mp.
4. 1 or 2
5. 3 and 4

CENTRAL

1. MeSH descriptor: [Arthroplasty, Replacement, Hip] explode all trees
2. total hip arthroplasty or total hip replacement or THA or THR
3. #1 or #2
4. quadratus lumborum block
5. #3 and #4
