# Supplementary figures and images for: Quadratus lumborum block for postoperative pain management in patients undergoing total hip arthroplasty: a systematic review and meta-analysis
Source: Hip Int. 2022 Jul 17;33(5):850–7. doi: 10.1177/11207000221111309 (PMC10486167; doi:10.1177/11207000221111309)

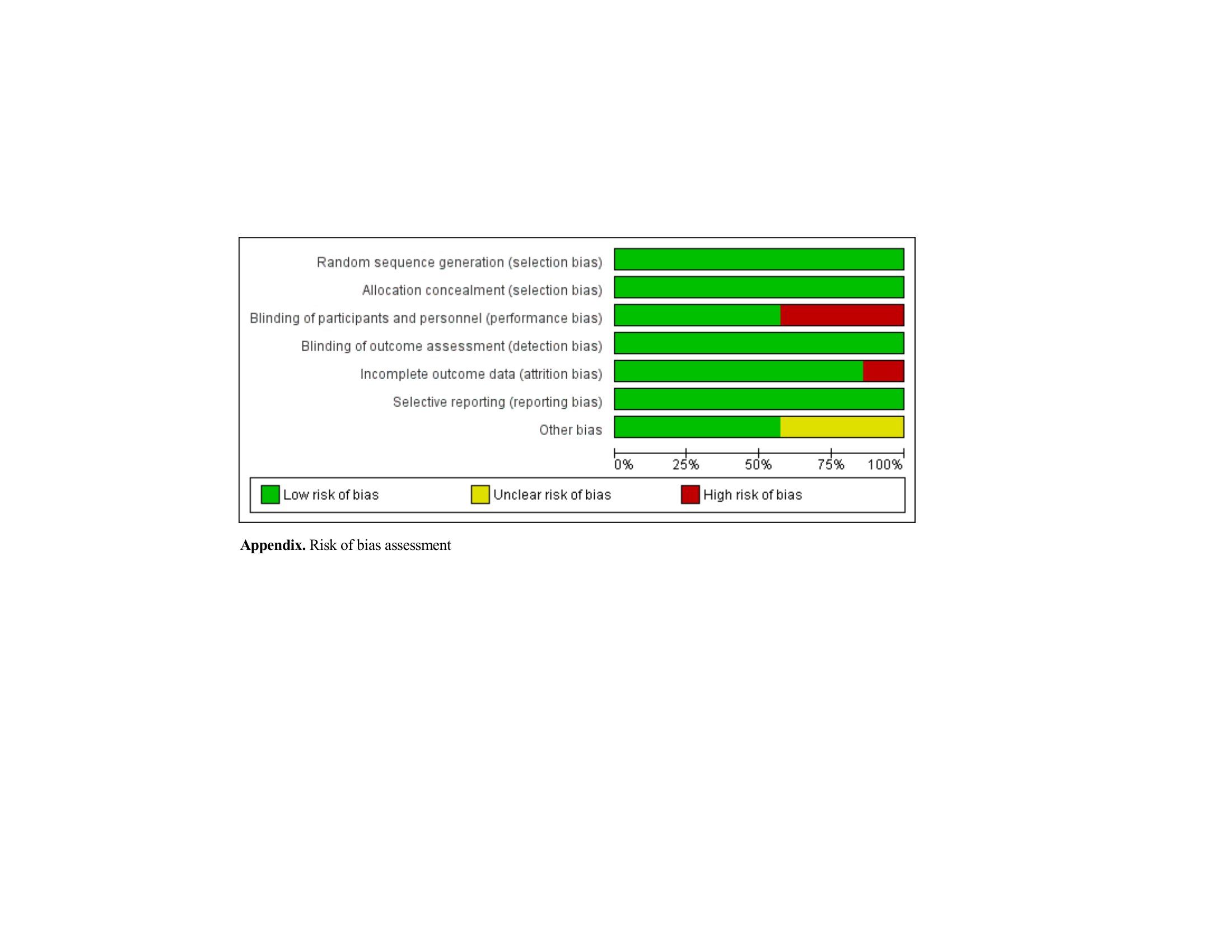

Supplement: sj-tiff-2-hpi-10.1177_11207000221111309 – Supplemental material for Quadratus lumborum block for postoperative pain management in patients undergoing total hip arthroplasty: a systematic review and meta-analysis [file sj-tiff-2-hpi-10.1177_11207000221111309.tiff]
